# Mucus Aberrant Properties in CF: Insights from Cells and Animal Models

**DOI:** 10.1016/j.jcf.2022.08.019

**Published:** 2022-09-16

**Authors:** Camille Ehre, Gunnar C. Hansson, David J. Thornton, Lynda S. Ostedgaard

**Affiliations:** 1 University of North Carolina at Chapel Hill, Department of Pediatrics, Marsico Lung Institute, Chapel Hill, NC; 2 Dept. Medical Biochemistry and Cell Biology, University of Gothenburg, Gothenburg, Sweden; 3 The Wellcome Trust Centre for Cell-Matrix Research, and The Lydia Becker Institute of Immunology and Inflammation, Faculty of Biology, Medicine and Health, University of Manchester, Manchester, UK; 4 Department of Internal Medicine, Carver College of Medicine, University of Iowa, Iowa City, IA

**Keywords:** Cystic Fibrosis, Mucus, Airway, Submucosal gland, Animal Models

## Abstract

Cystic fibrosis (CF), an autosomal genetic disorder caused by the dysfunction of the cystic fibrosis transmembrane conductance regulator (CFTR) protein, is characterized by mucus accumulation in the lungs, the intestinal tract, and the pancreatic ducts. Mucins are high-molecular-weight glycoproteins that govern the biochemical and biophysical properties of mucus. In the CF lung, increased mucus viscoelasticity is associated with decreased mucociliary clearance and defects in host defense mechanisms. The link between defective ion channel and abnormal mucus properties has been investigated in studies involving cell and animal models. In this review article, we discuss recent progress toward understanding the different regions and cells that express CFTR in the airways and how mucus is produced and cleared from the lungs. In addition, we reflect on animal models that provided insights into the organization and the role of the mucin network and how mucus and antimicrobial activities act in concert to protect the lungs from invading pathogens.

## 1 Introduction

Lung health relies on two main defenses: mucus and antimicrobials secreted from both surface epithelia and submucosal glands, as well as mucociliary transport to physically remove bacteria and other particles from the lung. MUC5AC and MUC5B, the two dominant airway mucins, have distinct roles in the lungs that have evolved to facilitate pathogen trapping and airway clearance [1–3]. MUC5B is produced at baseline in the lungs and MUC5AC is upregulated in response to a challenge, an infection, as well as a chronic lung condition like cystic fibrosis (CF) [4, 5]. In CF, dysfunction of the cystic fibrosis transmembrane conductance regulator (CFTR) causes reduced chloride (Cl^-^) and bicarbonate (HCO_3_^-^) secretion, which adversely affects the biochemical and biophysical properties of mucus, as well as bacterial killing. As a result of altered mucus properties, mucus detachment from the submucosal glands and the cell surfaces is compromised in CF lungs [6–9]. Several studies briefly reviewed herein used *in vitro* and *in vivo* models to shed light on the role played by CFTR function on mucus properties and how different regions and compartments of the lungs play a role in airway mucociliary clearance and innate defense.

## 2 Cells Expressing CFTR

As therapeutic approaches based on gene transfer and gene editing are rapidly evolving, it is important to identify the cell types and regions that express CFTR in healthy lungs. For more than a decade, CFTR was thought to be expressed predominantly in ciliated cells but recent technical advancements through transcriptome analysis at the single cell level revealed that ionocytes and secretory cells were the dominant cell types expressing CFTR in the lungs [10–12]. The distribution of these cells and level of CFTR expression varies throughout the lungs. Although ionocytes comprise <1% of the total surface epithelial cells, this cell type is responsible for more than half of CFTR transcripts in the murine lungs [11]. In both human and pig lungs, the number of high-CFTR-expressing ionocytes remains sporadic in the large airways and vanishes in the small airways, a region that is also devoid of submucosal glands [10, 13]. In the small airways, CFTR expression is present predominantly in secretory cells, a plastic cell type that produces MUC5B at baseline and serves as progenitor cells for ciliated and goblet cells. Hence, secretory cells can also produce MUC5AC. In both the human and mouse small intestine, CFTR is localized to goblet cells that produce MUC2; mouse models with defective CFTR develop a similar phenotype to the one of people with CF including bowel obstruction and bacterial overgrowth [14, 15]. Although the precise role of CFTR in secretory and/or goblet cells is not yet understood, the unexpected observations that mucin-secreting cells express CFTR suggests a much more complex and interwoven role of CFTR than previously assumed.

To optimize molecular therapies, it is critical to understand how CFTR dysfunction affects mucus properties or, more specifically, how low Cl^-^ and HCO_3_^-^ secretion alter mucin interactions, mucin secretion, and the organization of the polymeric network [16, 17]. Both mucin concentration and pH affect mucus biophysical properties. Decreased Cl^-^ secretion reduces fluid secretion, which increases mucus concentration, mucin entanglement, and adhesion to the cell surface [4, 9, 18]. Decreased HCO_3_^-^ secretion decreases air surface liquid (ASL) pH which compromises the expansion of mucins following granule exocytosis [19] and increases electrostatic interactions within the mucin network [20]. These biochemical changes worsen the viscoelasticity of CF mucus in the lungs and initiate mucus accumulation in other mucin-producing organs (e.g., pancreas, intestine, and sinuses) [21]. Treatment of CF human bronchial epithelial (HBE) cells with effective CFTR modulator compounds (e.g., combination of elexacaftor, tezacaftor, and ivacaftor) revealed that treatment facilitated the removal of mucus from the cell surface [8]. Similarly, extended cell washings on non-treated CF HBE cells effectively removed mucus, which is important for the success of gene transfer/editing for rare mutations not corrected by CFTR modulators. In the lungs, extended hydration can be achieved by inhalation of hypertonic saline, which is a standard treatment for CF [22, 23] that may improve gene targeting approaches.

## 3 Respiratory Tract Clearance

The MUC5B mucin is secreted by submucosal glands in the form of bundled strands and secretory cells localized to the airway surface epithelium. MUC5AC is secreted by surface epithelial secretory and/or goblet cells in the form of thin threads. The upper airways of pigs and humans have numerous submucosal glands that secrete both Cl^-^- and HCO_3_^-^-rich fluid and MUC5B [24]. Fluid flow pulls out the packed MUC5B mucin into linear strands that during the passage through the gland ducts assemble into thick bundled strands with over 1,000 linear MUC5B polymers [25]. Once on the tracheobronchial cell surface, these bundled strands are coated with MUC5AC threads that are secreted from the surface epithelial cells. In contrast to bundled strands that display a wide diameter in the 10-20 µm range, the diameter of mucus threads rarely exceeds a few micrometers [26]. The thin mucus threads are more efficient at collecting fluorescent nanospheres and bacteria than the bundled strands and aggregate into large formations prior to attaching to bundled strands [7,26]. In normal lungs, bundled strands, threads and collected material are transported cephalically by ciliary beating and are cleared from the lungs by coughing or swallowing [27].

Abnormal mucus properties are central to CF airway defects. In CF trachea, the mucus bundled strands sometimes fail to release from the submucosal gland ducts resulting in mucus aggregation and accumulation on the airway surface [6, 28]. Hyperconcentration of submucosal gland mucus increases cohesive forces, compromising the passage of mucus through the narrow diameter of the gland ducts [9]. In CF piglets, these bundled strands fail to move by a combination of attachment to the gland duct and/or to the surface cells [6, 7, 28]. Preventing Cl^-^ and HCO_3_^-^ secretion in non-CF pigs partially replicated this mucus defect [6, 28]. These studies suggest that loss of anion secretion by CFTR alters the properties of the submucosal gland mucus and impairs mucociliary transport.

## 4 Airways Defense

Mice, which naturally lack submucosal glands, require MUC5B, but not MUC5AC, for airway defense [29]. In larger mammals like pigs and humans, secretions from both the surface epithelium and the submucosal glands are important for airway function. Although *in vitro* cell cultures from both surface epithelia [30] and submucosal gland cells [31, 32] and tracheal explants have been used to model ion and mucin transport [33, 34] and local defense mechanisms [35], we still do not know the relative contributions of surface epithelia and submucosal glands to airway defense. To gain a deeper understanding of the role of submucosal glands *in vivo*, a pig model that lacked submucosal glands was developed to determine how loss of glands and glandular secretions would impact mucus transport and host defense. To create a ‘gland-less’ pig, the ectodysplasin gene (*EDA*) that encodes a protein necessary for duct and gland development was mutated to create a functional knock out [36]. This resulted in pigs (*EDA-KO*) that lacked submucosal glands throughout the conducting airway, which was confirmed by histopathology, gene expression, and proteomic studies. Excised tracheas from *EDA-KO* pigs were unable to secrete mucus strands. Mucociliary transport was abrogated in the proximal airways of *EDA-KO* animals, validating the importance of strand secretion to mucociliary transport. In addition, *EDA-KO* pigs had reduced bacterial killing in the proximal airways. This pig model was unable to eradicate bacteria introduced into the airways, consistent with absence of antimicrobials secreted from the glands. Thus, *EDA-KO* pigs lack two arms of host defense, mucociliary transport and bacterial killing. These results indicate that airway submucosal glands protect the lung by contributing to two critical respiratory host defenses, the production of bundled mucus strands necessary for mucociliary transport and the secretion of antimicrobials to kill invading pathogens [36].

## 5 Mucus in Non-Mammalian Models

As demonstrated above, the pig and other mammalian models have been critical in defining key aspects of mucin and mucus structure and function. However, there are still gaps in our knowledge of mucus structure and how dynamic changes in mucus composition and properties underpin the multiple roles which mucus plays in maintaining the healthy lung. In parallel, non-mammalian models (e.g., the *Xenopus tropicalis* tadpole) are being developed to investigate mucin, mucus and mucociliary biology [37]. The unique combination of conserved cell types (e.g., ciliated cells, mucin-secreting cells and ionocytes), genetic tractability, fast generation time and, unlike mammalian models, ease of access to mucin producing tissue on its external surface makes the tadpole model ideally suited for the study of mucus biology [37, 38]. The *X. tropicalis* tadpole has mucin-secreting cells in the skin surface, which produce the mucin, MucXS, that has structural similarity to mammalian polymeric, gel-forming mucins [39]. Functionally, MucXS underpins a host protective mucus barrier that overlays the tadpole skin surface and knockdown of MucXS causes a marked reduction in barrier thickness and loss of protection against infection [39]. Recent unpublished work from the Thornton laboratory has shown that the *X. tropicalis* tadpole model produces a distinct polymeric mucin, Muc5j, exclusively from a glandular structure at the head end (Dubaissi et al., unpublished). This mucin has similar domain organization to the mammalian polymeric mucin MUC5B, and forms strands analogous in morphology to MUC5B bundled strands produced by submucosal glands in the upper airways of the pig and human lungs (described above). The Muc5j-based strands are adherent and facilitate attachment of the tadpole to surfaces, moreover, material can bind along their length (e.g., bacteria-sized beads), suggesting similar function to the human and pig MUC5B-based bundled strands. The structural and functional similarities of the different types of mucin-based mucus forms produced by the *X.* *tropicalis* tadpole to those encountered on mammalian airway surfaces demonstrate the utility of this model system to discover new mucus biology relevant to the human lung in health and in CF. For example, targeting components that have roles in mucin/mucus production (e.g., ion channels (such as CFTR) that regulate ASL volume and impact mucin expansion would be informative.

## 6 Conclusions

The studies described in this review show that normal mucus biophysical properties and effective mucociliary clearance are dependent on functional CFTR [6,7,28]. In the last decade, the development and approval of highly effective CFTR modulators (e.g., Kalydeco and Trikafta) have significantly improved the quality of life of many people living with CF [40, 41]. Showing that CFTR rescue normalizes mucus further highlighted the role played by CFTR on mucus properties [8, 42, 43]. However, CFTR modulators fail to address the most severe mutations (e.g., Class I with defects in protein synthesis). Furthermore, many people taking CFTR modulators can develop side effects or suffer from severe lung damage acquired before therapy that may include sustained increased mucus production. Hence, it is critical to better understand mucus and its alterations in CF to identify novel pharmaceutical targets. Although more work remains to be done to fully elucidate the effects of decreased Cl^-^ and HCO_3_^-^ secretions on mucus, studies on cells and animal models have provided clues about the regions and the structures affected in CF, as well as the biochemical mucus alterations and impaired innate defense caused by CFTR dysfunction.

## Figures and Tables

**Fig. 1:**
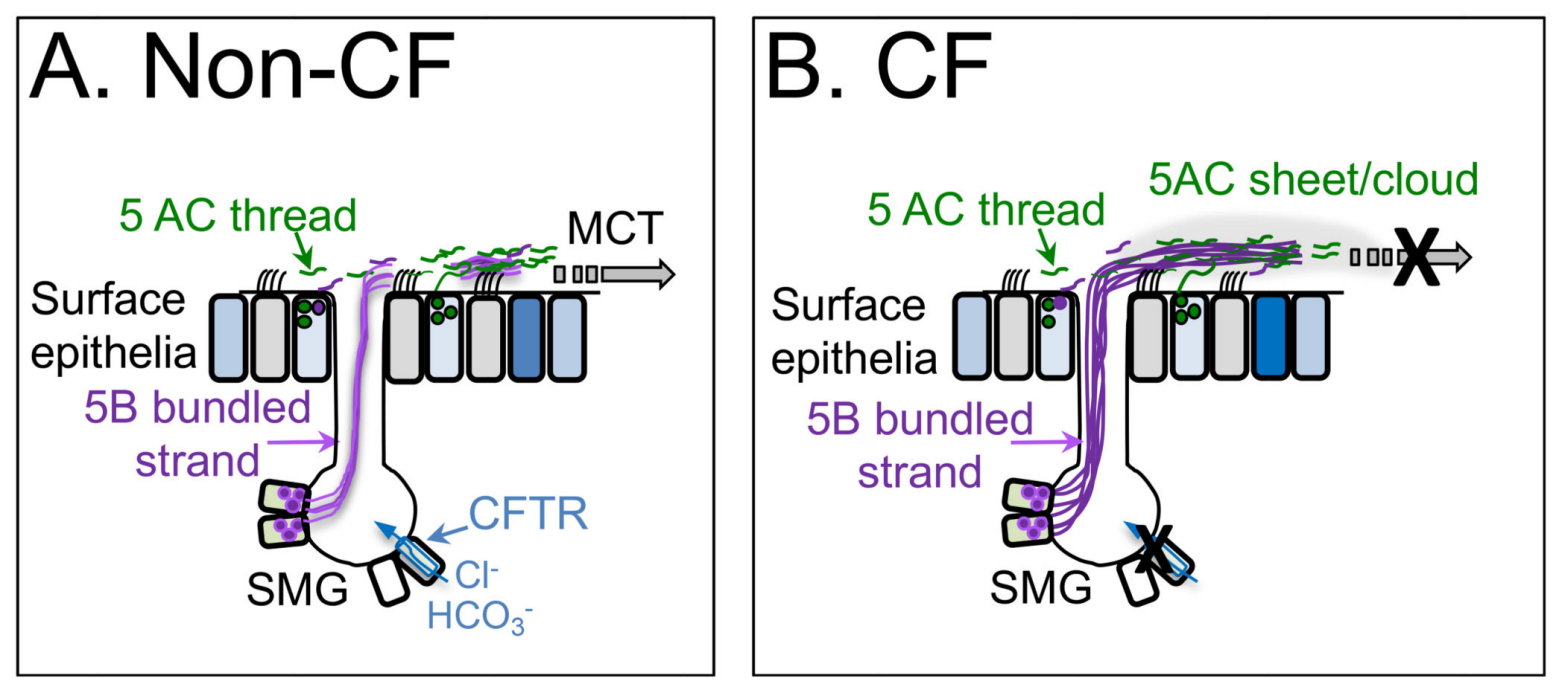
Mucin dynamics in pig trachea. A. Non-CF. MUC5B bundled strands (magenta) secrete from SMG onto airway surface, are covered with MUC5AC and MUC5B threads and are removed from airway by mucociliary transport (MCT) (grey arrow). CFTR is expressed in submucosal gland (indicated by blue arrow), in ionocytes (dark blue) and in secretory and goblet cells (light blue). B. CF. In the absence of functional CFTR, MUC5B bundled strands fail to release from SMG duct, are coated with MUC5AC threads and clouds, leading to accumulation on airway surface and decreased mucociliary clearance. Adapted from Ostedgaard et al, 2017, PNAS 114:6842-7 (Fig. 7).
